# AN IOT TROLLEY FOR LONG-TERM CARE FACILITIES TO PROVIDE EFFICIENCY AND REDUCE RISKS

**DOI:** 10.1093/geroni/igz038.1881

**Published:** 2019-11-08

**Authors:** Chia-Ching Chou, Ting-Ju Liu, Shih-Chung Kang

**Affiliations:** Smart Aging Tech, New Taipei City, Taiwan, Taiwan

## Abstract

One of challenges facing the long-term care facilities in Taiwan is the burden of the paperwork affecting nurses, which limits their time to look after the residents. Nurses usually estimate spending one quarter of their shifts with paperwork. The aim of this study is to develop a mobile care information system - Jubo IoT Trolley: a trolley with IoT vital-sign devices collecting and delivering timely care information to care professionals. Based on user-centered design (i.e., discover-define-develop-delivery), we conducted stakeholder interviews and rapid prototypes to zero in on the communication problem, and designed the IoT Trolley to support nurses in their daily workflow, facilitate vital-sign measurements at the bedsides, and collect the measured values to the cloud database automatically. Through design iterations, we have validated usability of the system in multiple care facilities. The result shows, with the IoT Trolley, the nurses can receive the senior’s critical vital status from the caregivers more promptly, provide instructions remotely and therefore, reduce potential care risks. Furthermore, the cloud analyzes the collected residents’ health data, the vital sign alerts can be sent to the nursing directors, so they can coordinate and intervene instantly. At last, this work demonstrates that through the technology, care qualities are improved, and care professionals can spend more valuable time with residents in the long-term care facilities.

